# Dosimetry in brain tumor phantom at 15 MV 3D conformal radiation therapy

**DOI:** 10.1186/1748-717X-8-168

**Published:** 2013-07-06

**Authors:** Larissa Thompson, Humberto Galvão Dias, Tarcísio Passos Ribeiro Campos

**Affiliations:** 1 Nuclear Engineering Department, Federal University of Minas Gerais, Belo Horizonte, Brazil; 2 Luxemburgo Hospital / Mario Penna Institute, Belo Horizonte, Brazil; 3 Nuclear Engineering Department, Federal University of Minas Gerais, Belo Horizonte, Brazil

## Abstract

Glioblastoma multiforme (GBM) is the most common, aggressive, highly malignant and infiltrative of all brain tumors with low rate of control. The main goal of this work was to evaluate the spatial dose distribution into a GBM simulator inside a head phantom exposed to a 15 MV 3D conformal radiation therapy in order to validate internal doses. A head and neck phantom developed by the Ionizing Radiation Research Group (NRI) was used on the experiments. Such phantom holds the following synthetic structures: brain and spinal cord, skull, cervical and thoracic vertebrae, jaw, hyoid bone, laryngeal cartilages, head and neck muscles and skin. Computer tomography (CT) of the simulator was taken, capturing a set of contrasted references. Therapy Radiation planning (TPS) was performed based on those CT images, satisfying a 200 cGy prescribed dose split in three irradiation fields. The TPS assumed 97% of prescribed dose cover the prescribed treatment volume (PTV). Radiochromic films in a solid water phantom provided dose response as a function of optical density. Spatial dosimetric distribution was generated by radiochromic film samples at coronal, sagittal-anterior and sagittal-posterior positions, inserted into tumor simulator and brain. The spatial dose profiles held 70 to 120% of the prescribed dose. In spite of the stratified profile, as opposed to the smooth dose profile from TPS, the tumor internal doses were within a 5% deviation from 214.4 cGy evaluated by TPS. 83.2% of the points with a gamma value of less than 1 (3%/3mm) for TPS and experimental values, respectively. At the tumor, measured at coronal section, a few dark spots in the film caused the appearance of outlier points in 13-15% of dose deviation percentage. And, as final conclusion, such dosimeter choice and the physical anthropomorphic and anthropometric phantom provided an efficient method for validating radiotherapy protocols.

## Introduction

Glioblastoma multiforme (GBM) is the most aggressive brain tumor. Two to three new cases per 10,000 inhabitants a year are reported in most European countries [1]. GBM corresponds to about 75% of all high-grade gliomas. Prognosis is poor, with average survival of just 12 to 16 months [2,3]. According to the American Cancer Society (ACS), about 22,340 malignant brain or spinal cord tumors will be diagnosed per year in the United States of America (USA), resulting in 13,110 deaths [4]. After a GBM diagnosis, the prognosis is poor [5-9] and median overall survival is less than one year [8], due to the uncontrollable infiltrative capability of such tumor [2]. Despite its uniformly lethal course, the survival of patients with GBM varies considerably [10]. In general, extensive portions of the brain are involved. GBM, depending on location, may be surgically removed. Radiotherapy prescribes daily absorbed doses of 1.8 *Gy* to 2.0 *Gy*, 5 days a week, reaching about 60 *Gy*[2]. Normal tissue capability to withstand radiation without deleterious effects depends on prescribed dose, irradiated tissue volume, tissue sensitivity, previous irradiation history, and personal radio sensitivity [11]. GBM local management is still a challenge, either on its initial stages or on recidivations [12]. In spite of GBM’s high degree of malignancy, recent studies show that survival rate has doubled in the last 30 years, due to advances in radiotherapy, surgery and chemotherapy. In the 1990’s, less than one in ten GBM patients would survive for more than six months. According to Beckford [13], half of such patients would survive for at least a year after diagnosis. That study stated that 17% of all primary brain tumors were GBM and less than 4% of those patients remained alive after 5 years.

Radiotherapy’s goal is to deliver a lethal accumulated dose on the tumor, while sparing adjacent healthy tissues from radiation’s detrimental effects [14]. In spite of being an effective treatment for GBM, radiotherapy’s deleterious side effects depend on the tumor’s oxygenation [15]. Brain tumors have a lower oxygen tension than normal surrounding cortex, making them more radio-resistant. Usually, survival rate is correlated to total radiation dose. Above 70 *Gy*, however, toxic effects on surrounding brain tissue overcome benefits from improved tumor control. Preserving the patient’s life quality is important in this context [15].

Precise evaluation of the doses at the tumor and surrounding regions is essential to maximize the treatment’s benefits. Thus, spatial dose distribution on either homogeneous or heterogeneous, solid phantoms is essential in order to validate radiotherapy planning, assuring quality and reproducibility of the exposures [16]. Dosimetry comprises determination of both quantitative dose and its spatial distribution, absorbed by several parts of the human body from internal or external radiation sources. Precise dosimetric evaluation is highly desirable, but challenging due to three factors: (1) possibility of several different exposure scenarios, even for a single spatial and temporal relationship between source and human body; (2) different exposure mechanisms due to distinct radiation physical properties, such as those of x-rays, gamma rays, electrons, positrons, neutrons, alpha particles, heavy ions or protons; (3) variations of density, anatomical shape and chemical composition present in the human body, which also changes with respiration and cardiac rhythm. Such factors make choosing representative parameters for the interaction between radiation and the human body a very complex problem [14]. The phantom used in this work is a static tool, however, the influence of movement and reproducibility were not prioritized. Due to GBM’s low survival rate treatment should be reevaluated, in particular the verification of the dose absorbed at the tumor provided by the current radiotherapy protocols.

Dosimetric film Gafchromic EBT2 was designed by International Specialty Product (ISP) for radiotherapy applications [17,18]. Gafchromic EBT2’s energy dependency is very weak in a wide range of radiation beam quality in radiotherapy [18]. EBT2 film’s chemical composition is equivalent to soft tissue ($Z_{\text{eff}\;\text{Film}}^{\text{Water}}=6.84$, compared to $Z_{\text{eff}\;\text{Soft}\;\text{Tissue}}^{\text{Water}}=7.3$). It provides good spatial resolution (< 0.1 *mm*) and may be submerged in water. It is, therefore, a good dosimetric tool for measuring percent depth dose (PDD) [19]. Gafchromic EBT2 has high sensitivity up to 80 *Gy* and, thus, may be used to measure absorbed dose in conformal radiotherapy, besides other modalities [20]. In fact, dose may be evaluated from 1 *cGy* to 10 *Gy* by readout on visible frequencies close to red and up to 40 *Gy* in the green light component [20]. Dosimetric films are able to register information with high spatial resolution while suffering little to none distortion in high-gradient regions, which are problematic for ionization chambers. Gafchromic EBT2 may be used as an alternative dosimeter for measuring dose depth percentage in megavoltage radiotherapy, for a wide range of energetic beams [19]. Despite the use of radiochromic films becoming routine in radiotherapy, spatial dose distributions in internal organs or even in tumor simulators have not been investigated, probably due to the reduced number of appropriated phantoms with internal organs available for conducting those experiments [21-23].

Two distinct spatial dose distributions, *e.g.* from TPS and experimental, may be compared based on quantitative parameters. The gamma index method, as presented by Low *et al.*[24] and Depuydt *et al.*[25], provided a dose-difference criterion, *ΔD*_*M*_, and the distance to agreement (DTA) criterion, *Δd*_*M*_, in order to evaluate and analyze an n-dimensional set of spatial distributed data comparing measured and calculated sets. The method incorporates dose and distance criteria, providing a numerical quality index that represents a level of agreement or disagreement in a tested region.

Previous studies show dosimetric measurement results by using radiochromic dosimeters in homogeneous and heterogeneous phantoms. Butson *et al.*[26] mentions investigation of radiochromic film for use in dosimetry in water phantoms as opposed to solid phantoms, and that the penetration rates of water into radiochromic film are measured in order to assess their effects on optical density. The effects of film orientation during irradiation in water were also tested. Butson *et al.*[26] included that the radiochromic film seems to be an adequate detector for dosimetry in a water phantom where high spatial resolution is needed [26]. Albertini *et al.*[27] performed measurements in an anthropomorphic phantom in order to investigate clinical relevant intensity modulated proton therapy (IMPT) treatment plans. Albertini *et al.*[27] had two goals: to assess plan accuracy in the presence of high heterogeneity and to measure plan robustness in the presence of treatment uncertainties. A phantom with five different tissue substitute materials, simulating different tissue types, and Gafchromic films were used. Their results showed excellent agreement between the calculated and the measured dose distribution: > 99% and 98% of points with a gamma value < 1 (3%/3 mm) for the 3D-IMPT and the DET plan, respectively. Nakano *et al.*[28] developed a study quantifying surface doses on several rectangular phantom setups and on curved surface phantoms for a 6 MV photon field using the Attix parallel-plate chamber and Gafchromic EBT2 film. Their results indicate the important role of the presence of bolus if the clinical target volume (CTV) is quite close to the surface, and demonstrates the suitability of Gafchromic EBT2 film for surface dose measurements in megavoltage photon beams. Horsfield [29] investigated EBT2 film irradiation with three different head and neck intensity modulated radiation therapy treatment plans using the *CIRS*^*TM*^anthropomorphic phantom using the gamma analysis criterion of 3mm5%, resulting in 90% agreement between the planned treatment and the measured values.

The main goal of this work is the evaluation of the spatial internal dose distribution in a anthropomorphic and anthropometric head and neck phantom with a GBM simulator inserted into the brain, at a central position, submitted to a three-field conformal radiotherapy 3D.

## Materials and methods

### Simulators, dosimeters and radiological imaging

A physical head and neck phantom developed by the NRI Research Group [21-23], including a GBM simulator, was used in our experiments. The GBM simulator was made from tissue equivalent material and incorporated into the anthropomorphic and anthropometric head and neck phantom’s brain. The equivalent tissues composition of head phantom was prepared based on a mixture of selected chemical materials, in stoichiometric proportions, that achieve the elemental human composition provided by ICRU-44. A set of chemical compounds and their weight proportions has been chosen in order to reproduce the elemental composition and mass density of the brain, bone, cartilage, muscle and skin tissues. All were prepared by synthetic materials. The required compositions were reached using water or jelly mixtures as the base material, based on carboxymethylcellulose and polymethylmetacrylate. The brain and the tumor tissues were prepared with agar-agar as base material [23]. That simulator weights 55 *g* and its volume is 20.05 *cm*^3^. It was inserted into the central region of the brain, affecting both hemispheres.

The chosen dosimeter was the radiochromic film Gafchromic EBT2 (lot number F020609). Such film has been used for radiation therapy dosimetry, being sensitive to daily exposure levels prescribed in current protocols [19]. Three groups of ten segments of Gafchromic EBT2, 1.0 × 1.5 *cm* each, were used in the calibration process. Three segments, two of 5 × 3 *cm* and one of 10 × 3 *cm*, were used for evaluating spatial dose distribution in the tumor. Those film samples, handled with surgical gloves, were cut and stored in an black envelope in order to protect them from any external damages such as scratches, moisture or light exposure.

The film samples were inserted into the physical phantom, positioned vertically in relation to the base of the skull, in a cross pattern, at the center of the GBM simulator. The following spatial orientations were adopted: two sagittal segments, anterior and posterior, and one coronal segment, from right to left. Film segments thus covered not only the tumor simulator, but also part of the adjacent equivalent tissue corresponding to brain. CT images of the physical phantom with the inserted film samples were taken at a Siemens SOMATON Emotion 6 system with 2 *mm* slices, 54.80 *mGy* dose, 108.8 *mAs*, 130 *kV*, multislice volume. Exact locations of the GBM simulator and the film segments inside the physical phantom were identified by radio-opaque markers. After the CT scans, the film segments were replaced by new ones, with the same dimensions, from the same lot and placed at the same orientation as the previous ones.

### Radiotherapy planning

Radiotherapy planning was based on the CT phantom images, digitized in DICOM mode. The image set was stored in a database. The prescribed dose was defined by the Radiation Therapy Oncology Group (RTOG) volume prescribed technique in which 97% of the dose covers the prescribed treatment volume (PTV). The irradiation target volume was delimited and a dose of 200 *cGy* was prescribed, so that all the target volume was covered. Radiotherapy planning on the physical phantom was performed by CAT3D for Win32 software version 7.08e. Planning was based on the brain tumor protocol suggested by the Radiotherapy Service of Luxemburgo Hospital, Belo Horizonte, Brazil. The film segments inserted into the physical head and neck phantom, designated as coronal (C), sagittal-anterior (SA) and sagittal-posterior (SP) received a single fraction of 200.0 *cGy* as target dose. The region of interest received the irradiation of three fields: antero-posterior 6.0 × 7.0 *cm*, lateral-right 6.0 × 7.5 *cm*, and lateral-left 6.0 × 7.5 *cm*. The technique was 3D isocentric conformal radiotherapy, with gantry angle of 0° antero-posterior, 270° lateral-right and 90° lateral-left, with wedge W30 filters at the lateral fields. Monitor units were 93.4, 90.3 and 88.8 *cGy* at the antero-posterior, lateral-right and lateral-left fields, respectively. For calibration purposes, temperature and pressure inside the irradiation room were measured, and a calibration coefficient *KPT* = 1.1297 determined. The particle accelerator was also calibrated so that 1 *cGy* would correspond to 1 Monitor Unit (MU).

Dosimeter calibration was performed using a water phantom, an acrylic box filled with water, developed by the NRI Research Group. Such phantom has 4 *mm* walls and external dimensions of 304×308×308 *mm*. At the center of the box there was an adjustable support. Alongside its height there were ten square holes, to support ten horizontal brackets of 5 × 5 × 100 *mm*. Such brackets held the film pieces during the calibration process. The distance between holes was 2.0 *cm* center-to-center, lowest hole 6.5 *cm* from the bottom and highest hole 4.5 *cm* from the top. The brackets have cuts to hold the film pieces. The film piece on the topmost bracket was covered by 3.0 *cm* of water, ensuring electronic equilibrium. Exposure was measured by ionization chamber and dose converted at the film samples’ positions, generating the percent depth dose profile. The calibration of the 15 MV beam was prepared based on the Technical Report 398 [30]. The uncertainty from dose calibration is a constant value independent of the dose.

### Percent depth dose evaluation and simulator irradiation

Percent dose depth (PDD) profile was generated using the water phantom. At this point, 30 film pieces were set aside in three groups of ten segments each. Each film group was positioned, affixed in depth on the ten horizontal brackets inside the water phantom. A rectangular radiation field of 10 × 10 *cm* was standardized. The isocenter of the film piece set was at 100 *cm* of the accelerator’s isocenter (source-surface distance - SSD). The planned dose was divided in three distinct ranges: 224.0 to 109.0 *cGy* (group I), 111.5 to 54.3 *cGy* (group II), and 55.3 to 26.9 *cGy* (group III). Irradiation protocol was repeated for each group.

Exposure of the dosimeter set on the water phantom and on the physical phantom itself were both performed at the Radiotherapy Service of Luxemburgo Hospital / Mário Penna Institute. The linear accelerator used for calibration and irradiation procedures was Linac Saturne 2 CGR, 15 *MV*, *cGy*/*MU* = 1.000, for 6 and 15 *MV*, with nominal dose rate of 200 *MU*/*min*.

The phantom was held by a styrofoam support. Positioning followed the references from CT phantom images and the external, physical markings on the phantom’s outer surface, reproducing the orthogonal laser orientation captured at the moment of the CT scan (7.2 *mm* to the left, 6.8 *mm* backwards and 0.0 *mm* towards the top of the head). Exposure was performed in the convolution - pencil beam mode, 8 × 8 *mm*. The films were placed in a cross-shaped pattern, perpendicular to each other. The three radiation beams were also applied at 90° each other. However, they were applied with an angular tilt deviation from the film plane to the beam isocenter, in order to have the beam crossing the film while avoiding being parallel to the interface between film and brain. The isocenter of the film was defined based on small sphere markers made of lead.

### Digitalization and dosimeter readout

Digitalization of the exposed film samples was performed with a regular table scanner, HP Scanjet G4050, operating in transmission mode. It occurred 24*h* after exposure, ensuring that the film was fully self-developed. The scanner was warmed up for half an hour, in order to stabilize the temperature of its light bulb. Five sweeps of unexposed Gafchromic EBT2 film, and one of overexposed radiography film, were performed in order to prevent possible noise and artifacts, as suggested by [31]. Overexposed film, unexposed film, and the irradiated film samples were then digitalized as slides, using the scanner’s Transparent Materials Adapter (TMA), with the following settings: 300 pixels per inch (ppi), RGB (Red, Green, Blue) mode, 48 bits, 16 bits per color. The resulting files were in TIFF and JPEG format. The 30 calibration film samples were scanned together at the center of the scanner. After that, the three irradiation film samples were also scanned.

The digitalization process resulted in six groups of images: one unexposed film sample, one overexposed film sample, three film samples irradiated at the physical head and neck phantom (C, SA and SP), and three groups of ten film samples irradiated at the water phantom, for a total of 35 film samples. Digital images were analyzed on the ImageDig software package [32]. Color intensity in RGB mode was obtained, assuming a range from 0 to 255 on each component. Red and Green components from the film samples were thus measured. Mean value of nine measured points and standard deviation were calculated for the Red (*m*_*Red*_ and *σ*_*Red*_) and Green (*m*_*Green*_ e *σ*_*Green*_) components. The RGB pixel values were used rather than the physical quantity I.

### Optical density, standard deviation and dose response

Film exposure to radiation promoted darkening on red and green components. Optical density (OD) values and respective standard deviation (*σ*_*OD*_) were calculated from measurements of color components’ intensities. A film sample’s OD on a given component was evaluated as [31,33]:

(1) $$\text{OD}=\log_{10}\frac{I_{0}}{I},$$

where *I*_0_ is the RGB intensity on unexposed film, and I is the intensity on irradiated film. m(*RGB*_*unexposed*_) and m(*RGB*_*irradiated*_) were used as *I*_0_ and *I*, respectively. OD’s standard deviation is given by [31,33]:

(2) $$\begin{array}{cc} \sigma_{\text{OD}}(D)=\frac{1}{\ln10} & \sqrt{\frac{\sigma {\left(\text{RG}B_{\text{unexposed}}\right)}^{2}+\sigma {\left(\text{RG}B_{\text{overexposed}}\right)}^{2}}{{\left(m(\text{RG}B_{\text{unexposed}})-m(\text{RG}B_{\text{overexposed}})\right)}^{2}}+\frac{\sigma {\left(\text{RG}B_{\text{irradiated}}\right)}^{2}+\sigma {\left(\text{RG}B_{\text{overexposed}}\right)}^{2}}{{\left(m(\text{RG}B_{\text{irradiated}})-m(\text{RG}B_{\text{overexposed}})\right)}^{2}}}. \end{array}$$

*m*(*RGB*_*unexposed*_) and *σ*(*RGB*_*unexposed*_) are average and standard deviation of unexposed film, *m*(*RGB*_*overexposed*_) and *σ*(*RGB*_*overexposed*_) are average and standard deviation of overexposed radiographic film, and *m*(*RGB*_*irradiated*_) and *σ*(*RGB*_*irradiated*_) are average and standard deviation of the irradiated film’s color components, either red or green. Equation (2) was used in order to calculate standard deviations for each color component, red or green, individually.

Dose response was obtained assuming a linear relation between dose and optical density, within the exposure range on the experiment. The standard deviation for dose was as prescribed by technical report 398, and the standard deviation for optical density was calculated as in Eq 2. A linear regression between optical density and calibration dose was calculated. OD behaves linearly in relation to dose, even though *I*_0_/*I* behaves exponentially. The relationship between optical density and dose was evaluated for each of the three 10-sample calibration groups, and for the unexposed film. Calibration curves were generated by OriginLab Data Analysis and Graphing Software 6.1 for each component, red and green, independently [34].

### Absorbed dose on the GBM simulator

After digitalization and readout of the radiochromic film samples, as described above, absorbed dose on the GBM simulator was evaluated. Optical densities registered on C, SA and SP film samples were evaluated at pixels of the images. From dose versus optical density responses, for each dose interval, optical densities were converted to doses and plotted. Dose spatial distribution was thus generated for C, SA and SP film samples.

### Gamma-index and dose deviation percentage evaluation

Three sets of data and associated matrices *M*_*c*_(*i*,*j*), *M*_*m*_(*i*,*j*) and *M*_*t*_(*i*,*j*) were selected, two of them related to spatial dose distribution and one referring to a target organ position. Those matrices are representative of the three bidimensional segments taken from: a section of the therapy planning system (TPS), a section of a Gafchromic film detector, and a part of the photographic representation of the tumor, respectively. The two dose distribution matrices provide the calculated dose *D*_*c*_(*i*,*j*) from TPS and the measured dose *D*_*m*_(*i*,*j*) estimated by the Gafchromic films at red component. The target organ position matrix *M*_*t*_(*i*,*j*) was obtained from digitalization and processing of a photography of the tumor section where the radiochromic film was placed into. Each element of the *M*_*t*_(*i*,*j*) matrix holds one bit, zero or one, representing whether the corresponding position belongs or not to the target organ. The reference size length and width of the bidimensional segments of the calculated and measured dose distributions were also provided. The quality index *γ*(*r*_*m*_) corresponding to the measurement point *r*_*m*_, in the evaluated tumor area, with modular distance between *r*_*c*_ and *r*_*m*_ positions, and dose differences *D*_*c*_(*r*_*c*_) and *D*_*m*_(*r*_*m*_) obtained from *M*_*c*_ and *M*_*m*_ matrix data were evaluated. The gamma-index method and its criteria of acceptance were applied as described in the literature [24,25], but only to the tumor target area. The passing criteria adopted on this experiment were *ΔD*_*M*_ = 3% and *Δd*_*M*_ = 3 mm, as suggested by Low *et al.*[24]. The comparisons were repeated for all measurement points *r*_*m*_, and a bidimensional representation of the gamma-index was obtained. Dose deviation percentage between TPS and measured data was also evaluated at the tumor, limited to the coronal section.

### Dose volume histogram

Despite being a 2D representation of a 3D dose distribution, a dose volume histogram (DVH) was obtained from data at the tumor section. Its volume corresponds the portion of the film representing the tumor, a slice where radiation is absorbed. The histogram accounts for the portion of the volume that receives a specific dose or more. DVHs may be constructed as differential DVH’s and cumulative DVH’s. They were created by first determining the size of the histogram dose bins, arbitrarily defined as intervals of 14 cGy. The elemental volume was taken as 1 *mm*^3^.

## Results

### Simulators

**Figure 1:**
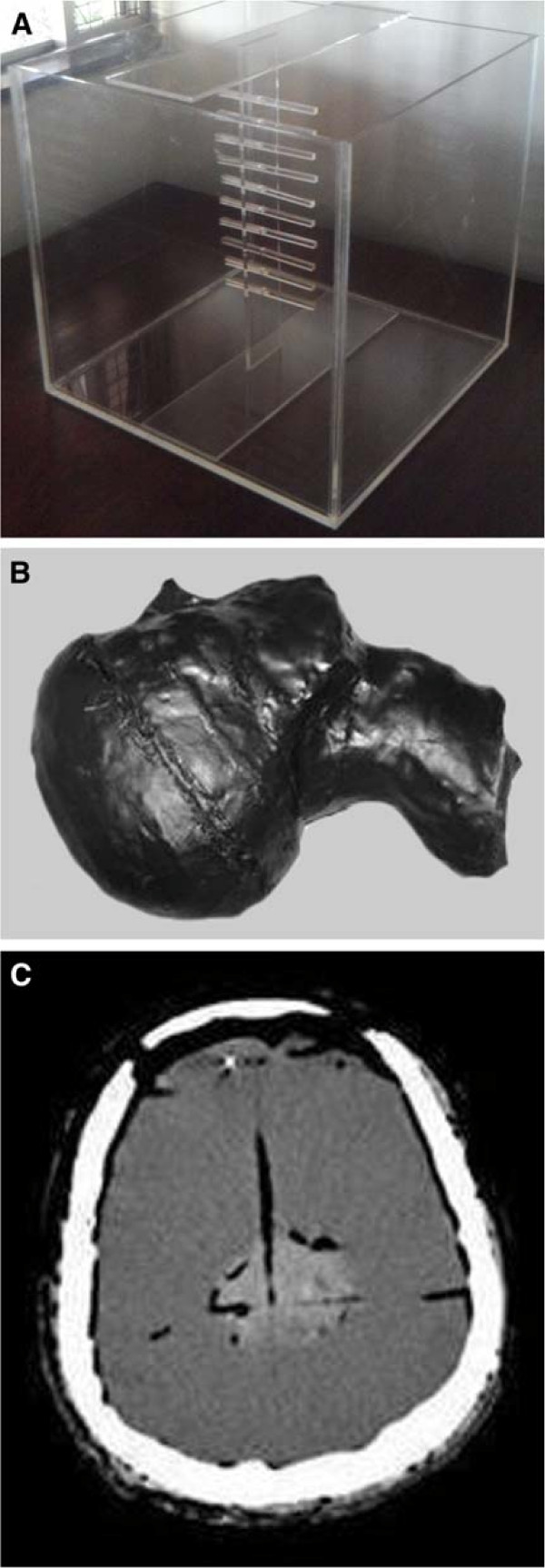
**Simulators. (A)** water phantom for calibration, **(B)** physical head and neck phantom, **(C)** CT section of the physical phantom with GBM simulator and film samples inserted.

Figure 1 shows the water phantom, the physical head and neck phantom, followed by the image of a CT section of the phantom. The cross pattern positioning and spacing of the film samples inside the head and neck phantom is also depicted.

### CAT3D radiotherapy planning on the physical phantom

**Figure 2:**
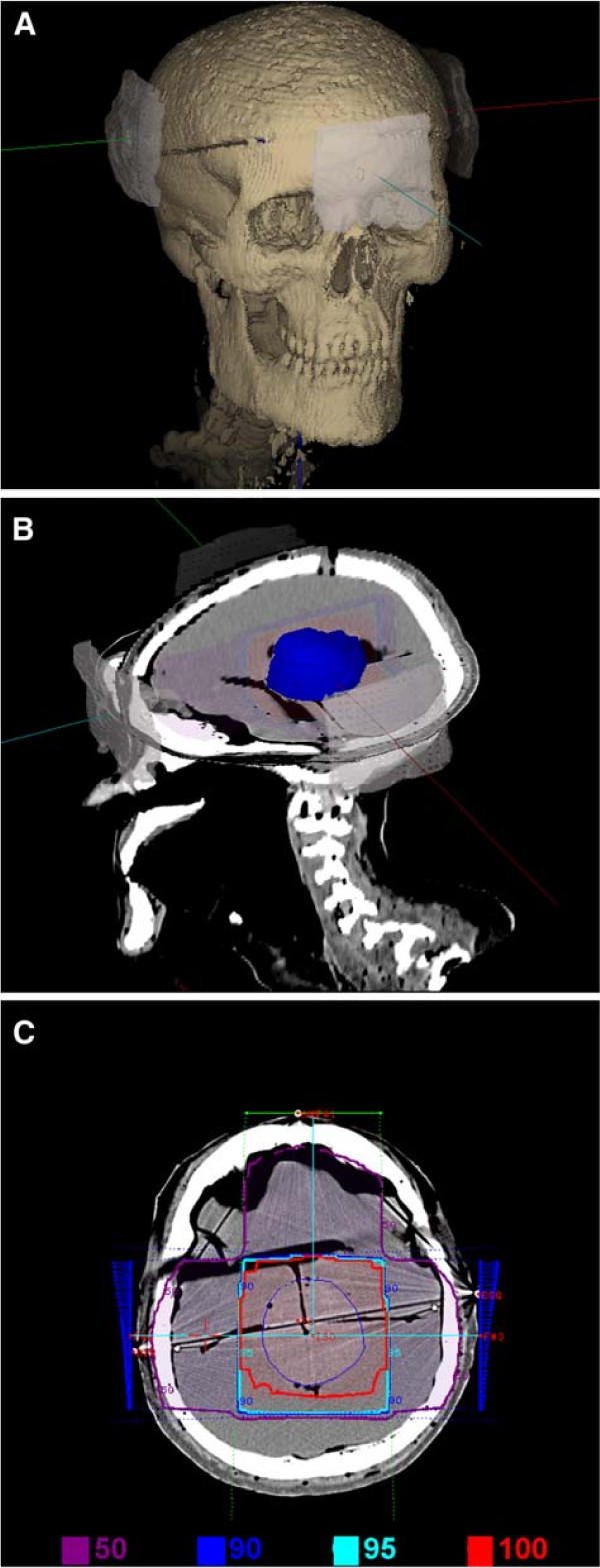
**Radiotherapy planning details. (A)** region of interest (cranial) and the three irradiation fields (antero-posterior, lateral-right, lateral-left), **(B)** region of interest and target delimitation (tumor simulator) and **(C)** the three fields (40% of the dose anterior, 30% right and 30% left), and respective isodose levels and two wedge filters.

Figure 2 shows details of the radiotherapy planning for the physical head and neck phantom. Projections of the three fields, anterior-posterior, lateral-right and lateral-left, are shown superimposed to the 3D-reconstructed images of the phantom’s skull on Figure 2A. Figure 2B shows the isodose curves planned on the region of interest, with the target (tumor simulator) delimited. Figure 2C shows the three irradiation fields, taken on the coordinate axes. The planning details are also presented: 40% of the dose (80 *cGy*) on the anterior-posterior field, 30% of the dose (60 *cGy*) on the lateral-right field and 30% of the dose (60 *cGy*) on the lateral-left, covering the tumor simulator with 100% of the dose. Isodose curves are shown for 100% of the dose (200 *cGy*), 95% of the dose (190 *cGy*), 90% of the dose (180 *cGy*) and 50% of the dose (100 *cGy*). Tumor simulator limits and the two bilateral wedge filters are also shown.

**Figure 3:**
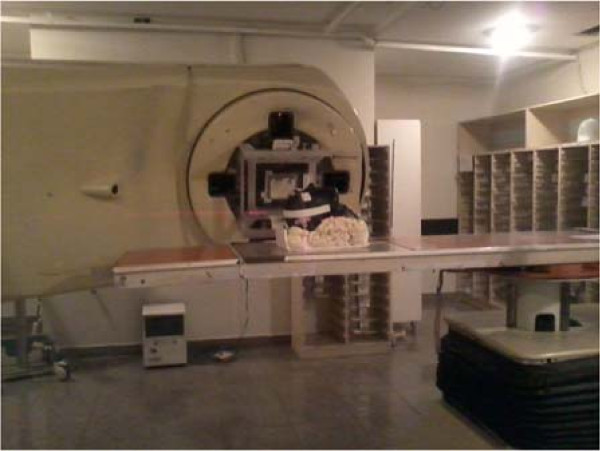
**Phantom positioning in relation to the accelerator gantry. **Planning and beam positioning indicated, based on the CT images.

Figure 3 shows phantom positioning and the irradiation fields. CT image and the physical markings on the phantom were used as references.

### Dose versus response relation

Curves for optical density as function of dose were obtained from optical density values and their standard deviations for each component, red and green, in RGB. After line fitting, the calibration curve representing dose as function of optical density on exposed film was expressed as:

(3) D=A·OD+B,

where *A* is line inclination and *B* is the intersection with the ordinate axis.

**Figure 4:**
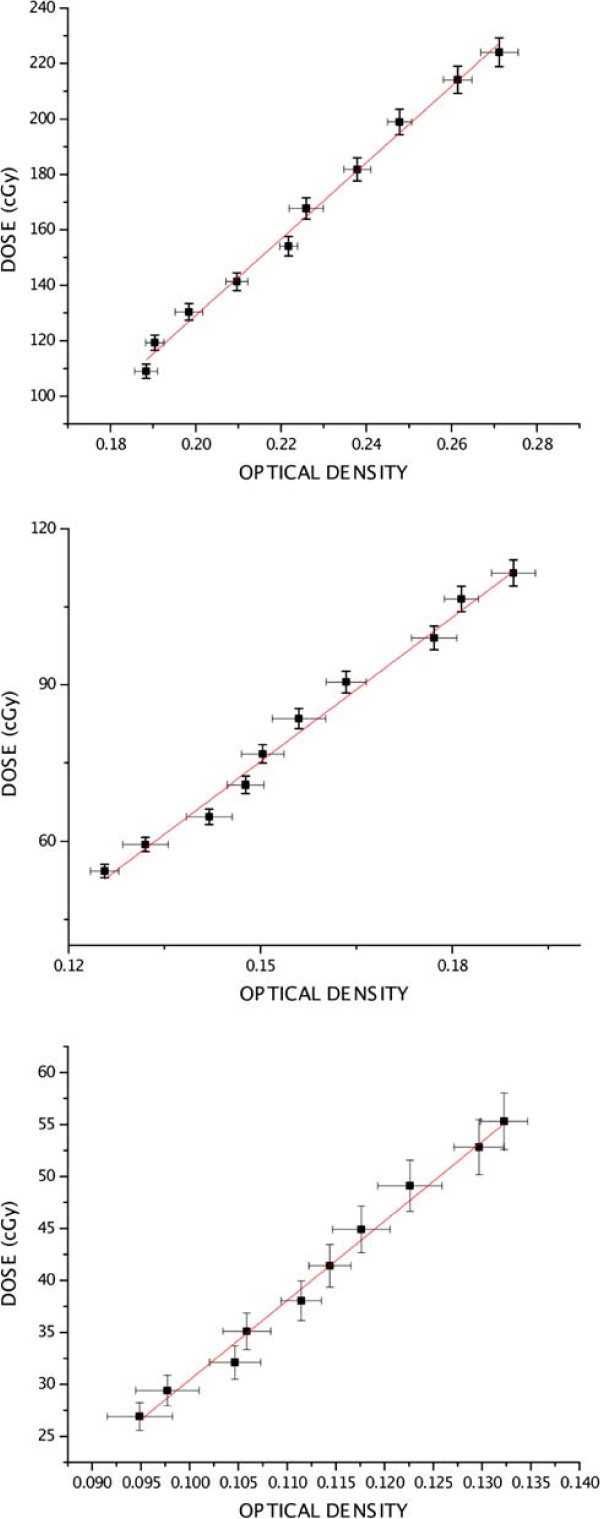
**Calibration curves, red component. **Calibration curves for the three film groups and line fitting, red component.

**Figure 5:**
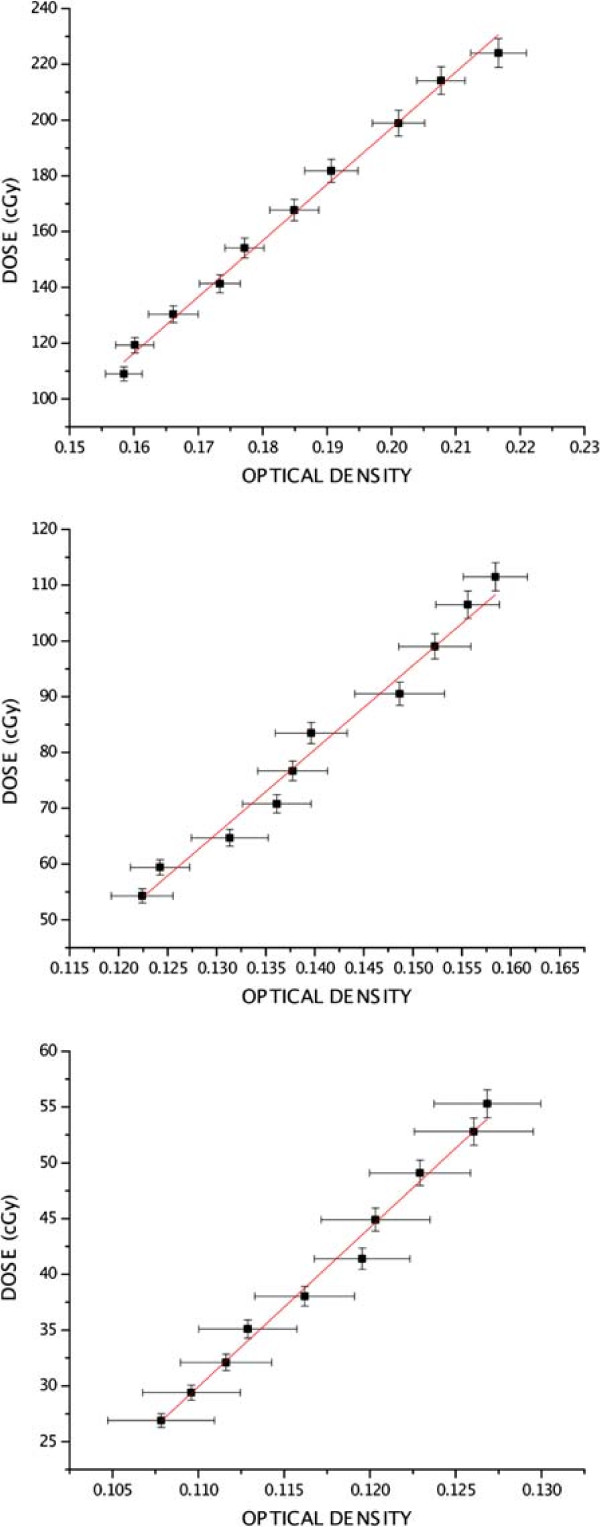
**Calibration curves, green component. **Calibration curves for the three film groups and line fitting, green component.

Figures 4 and 5 show film calibration curves for red and green components, respectively. A linear regression between dose and OD was calculated. After line fitting, parameters for the dose as function of optical density were found, for red and green components.

### Absorbed dose on GBM simulator

**Figure 6:**
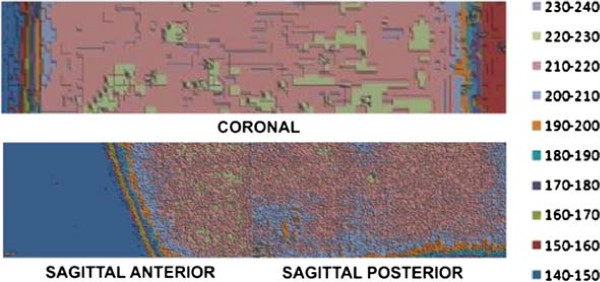
**Spatial dose distribution, red component. **Spatial dose distribution measured on red component, on C, SA and SP films, surface-contour mode.

**Figure 7:**
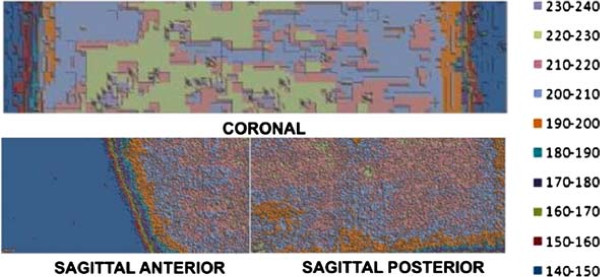
**Spatial dose distribution, green component. **Spatial dose distribution measured on green component, on C, SA and SP films, surface-contour mode.

**Figure 8:**
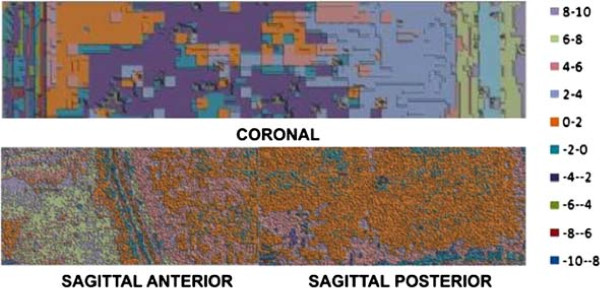
**Percent deviations, red and green components. **Percent deviations of measured doses from red and green components, on C, SA and SP films, respectively.

The spatial distribution of absorbed dose in the GBM simulator inside the physical head and neck phantom was obtained by applying optical density values into dose-response function. Dose distributions from red and green components on films C, SA and SP generated 3D graphs showing dose as function of position. Figures 6 and 7 show those spatial distributions, red and green components, respectively. Figure 8 shows the percent dose deviations measured on red component compared to green component, on C, SA and SP films. The small deviation values from calculated dose and red and green components show that both may be used for dosimetric evaluations in the range from 20 to 230 cGy.

### Gamma-index and dose deviation percentage results

**Figure 9:**
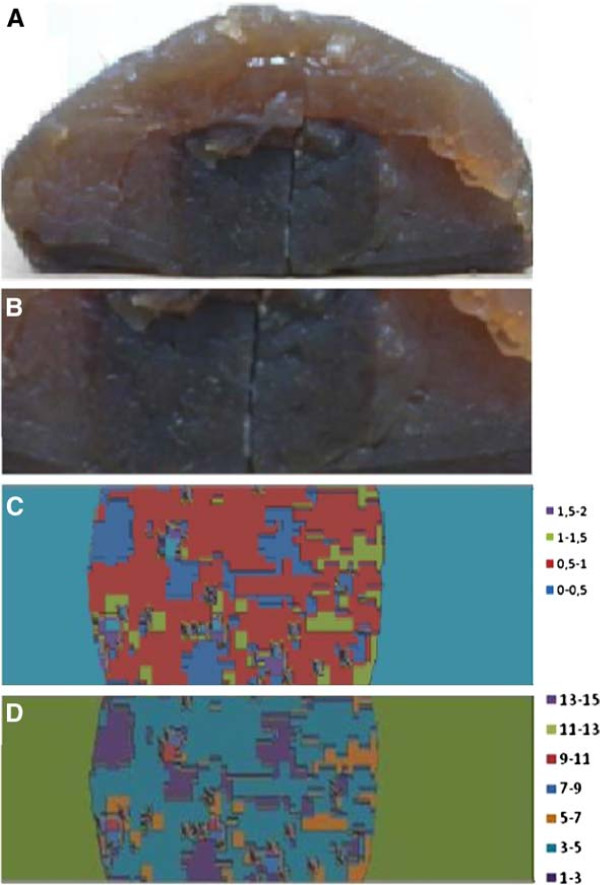
**Gamma-index and histogram results. (A)** Cross-section of part of the synthetic brain and the tumor area; **(B)** projection of the tumor section in which the coronal film was placed; **(C)** gamma-index values at tumor area; and **(D)** dose deviation percentage, from measured and TPS doses at tumor in the coronal section.

Figure 9C shows the results of gamma-index analysis applied to the tumor area. Values lesser than unit correspond to points from the image that have passed on the gamma-index criteria. All those *r*_*m*_ points, measured by radiochromic film enclosed by the tumor, provide limit points *r*_*c*_, in the calculation matrix produced by TPS, whose distance *Δr* and dose difference *ΔD* from *r*_*m*_ are enclosed by the 2D ellipsoid defined by *ΔD*_*M*_ and *Δd*_*M*_ parameters.

Figure 9D shows dose deviation percentage from *D*_*c*_ and *D*_*m*_ at the tumor area. Almost all points are in the two deviation intervals 1-3% and 3-5%. A few areas are in the 9-11% interval, and there are some stray points where film darkness provided the appearance of dose deviations in the higher interval, 13-15%.

### Dose volume histogram

**Figure 10:**
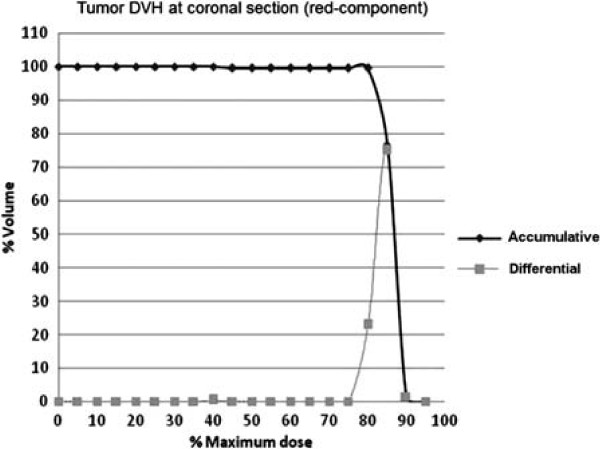
Accumulative and differential dose-volume histograms at tumor area in coronal section.

Figure 10 presents cumulative and differential dose-volume histograms (DVH’s). The DVH’s summarize dose distributions at the section of the film corresponding to the tumor, relating volume percentage to maximum dose percentage. The volume implicit in the DVH analysis corresponds to the arbitrary tumor target section in which the radiochromic film was placed. In the differential DVH, column height indicates the volume of the structure which received a dose given by the volume bin. In the cumulative DVH, column height of the volume bin 85% represents the percentage of the structure volume which received a dose greater than or equal to 214.2 cGy. No volume greater than 1 *mm*^3^ received 90%, which corresponds to doses greater than or equal to 239 cGy.

## Discussion

The main original characteristics of this study, compared to previous works [26,27,28,29], are the use of an anthropomorphic and anthropometric phantom which takes into account the heterogeneity of the human body and the adoption of a protocol, 3D conformal radiotherapy, actually used in a reference hospital for treatment of patients. In contrast with more controlled experimental setups, our experiment aims to reproduce realistic conditions, even with many possible sources of error, and improving the evaluation of actual internal doses at the brain. Those insights may be used for improving the quality of radiotherapy treatment.

The phantom was built with the head tilted in relation to the neck (hyper extended), so that it could be also utilized for head and neck dosimetry. Despite that, irradiation of the synthetic tumor at the center of the brain was performed equivalently to the human case. A support for the head was used so that its position relative to the beam orientation and entry points was as prescribed by the three field 15MV conformational radiation therapy protocol used for brain tumors. The size of such support required some lowering of the bed, in order to achieve correct beam. On this experiment, the influence of phantom movement was not considered. The experiment may be repeated, but there are no means of ascertaining the exact same dosimeter positioning due to phantom-film physical manipulation, therefore each experiment is unique and cannot yield exactly the same results.

The low attenuations of the 15 *MV* beam at a 20 *cm* depth required use of three calibration levels, corresponding to three distinct exposure ranges, measured by the three sets of ten film segments each. It was possible then to obtain a dose response versus optical density for the 26 to 224 *cGy* interval. In the present work, however, EBT2 film was selected and doses were measured on two components, red and green. Calibration with Gafchromic EBT2 film samples inside the water phantom followed similar protocols available in the literature [31,33]. Calibration curves have shown that optical densities increased linearly with dose for both components at the studied dose interval.

Dose distribution on coronal film, red component, showed an absorbed dose of 210 to 220 *cGy* (105 to 110%) on most of the film area, which is not statistically different from the prescribed dose value (PD) of 200 *cGy*±5*%*. Hot regions with 220 to 230 *cGy* (110 to 115% of the PD) were observed, however, and even a few hotspots with 230 to 240 *cGy* (115 to 120% of the PD) were present. The same pattern of spatial dose distribution was observed on the green component. Such hotspots were not identified on the radiotherapy planning. Red and green components provide dosimetric evaluations in the range from 20 to 230 cGy. Despite the fact that those responses are from different wavelengths, both come from the same dosimeter and are not independent. Variation between dose measurements on red and green components went from -4 to 6%, showing that both results were equivalent.

Dose distribution on sagittal-anterior e sagittal-posterior film samples, red component, showed that absorbed dose was between 210 and 220 *cGy* (105 to 110% of the PD). Those values are within the prescribed range. There were, however, regions with doses from 220 to 230 *cGy* (110 to 115% of the PD). The same spatial dose distribution pattern was observed on the green component, with deviations from 0 to 2%. Those small deviation values from calculated dose and red and green components show that both may be used for dosimetric evaluations in the range from 20 to 230 *cGy*, and one may be used to cross-validate the other. Sagittal-anterior film showed a cold region within 140 to 150 *cGy* (70 to 75% of the PD). Such area corresponds to the film segment between isodose curves of 60 to 80%.

Isodose curves generated during planning were smooth, covering the tumor with 100% of the prescribed dose (Figure 2C). Values measured show a non-uniform dose on film samples, varying from 105 to 115% PD, and some hotspots with 115 to 120%. Such non-uniform dose distribution, compared to planned isodose curves, is due to the heterogeneous features of the physical phantom, including bone, brain and the GBM simulator itself. Such structures, made of distinct tissues with different densities and chemical compositions, create variations in radiation absorption as compared to that of water.

Figure 8 presents the dose percentage deviations from red to green components. In the Sagittal Posterior (SP) film, there are deviations from -4 to -2% in a small area. Large areas in SP and Sagittal Anterior (SA) films present discrepancies of 0 to 2%. SA film presents areas from 4 to 6% deviation. In the Coronal (C) film, there are large areas of -4 to -2% and of +2 to +4% deviations. It is unclear whether the spatial representation of such discrepancies is meaningful. Film response at green and red are in distinct wavelengths and intensities. The red response provided higher response than green. In this experiment, the OD response interval for green was up to 0.217 while in red, it was 0.280. This means that the red component is more sensitive than the green one.

Application of the gamma index criteria to a target area of interest represented by the tumor was indicated. It was not applied to all of the bidimensional plane of measured data set compared to the TPS, since many discrepancies correspond to highlights due to high dose gradient regions at the interface of the irradiation window, as well as imprecise superposition between measured data and isodose TPS distributions, due to the lack of internal reference points. However, tumor identification was easily obtained from CT image, equivalent-tissue differences and physical tumor parameter differences, such as color and texture on the phantom. The main benefit from applying the gamma-index method to the tumor is comparing actual measured doses to calculated ones.

As demonstrated by the cumulative DVH, the tumor received a homogeneous dose. Since the cumulative DVH appeared as a horizontal line at the top of the graph, 100% of the volume received 82% of the maximum dose, 206.6 cGy, and no volume received higher than 90% of the measured dose. Dose spots present on the film alter the measured DVH from 90% up to 100%.

The value of 100% was normalized to the 97% of the overall prescribed dose of 200 cGy, which means that 100% corresponds to 206.2 cGy. The TPS provided a volume in which the tumor is enclosed with a dose at list superior to the 206.2 cGy. The DVH histogram for the whole tumor volume from TPS provided a maximum dose of 104% and an average dose of 102.3%, which means 210.9 cGy. Therefore, the tumor volume is covered by 214.4 cGy evaluated by the TPS. The precision error of the CAT3D TPS program provided a variation error to different protocols. The closest protocol tested to our experiment was a four two-opposite size irradiation field, providing a error of -3%. Therefore, it seems that the TPS may sub-estimate the dose up to -3%, which means the tumor dose shall be close to 220.8 cGy [35].

Few physical dark spots on the irradiation film responsible to high dose at the pixel level at the resolution of 0.1 mm per pixel. Since they are physical optical response of the crystals on the film, they were enclosed to the dose response measurements in the same resolution level. One can question if those spots provide precise dose responses in such resolution. Indeed, the film is intended for dose measurement at spatial resolution up to 0.1 mm, which it is the limit that our experiment is performed. At a higher spatial resolution the light transmission becomes increasingly noisy and the dose measurement of tiny pixel is less accurate. However, low dose spots were not observed. Also, Devic *et al.*[31] suggested that the averaging of the film response and a Wiener filter application are requirements to remove the“bad” pixels. However, on this article, the data were shown at it is. There is the possibility that those pixels are not representative of the dose profile. Further investigation shall confirm the high dose spots at the high resolution level and may be removed of the measurement set of data.

The uncertainty determinations follow ISO guide as suggested by [30]. There are many uncertainties that affect the applied method. Table 1 presents a summary of the variables that may influence the reproducibility of the dose measurements at the high energy photon beam condition, the head and neck phantom and the radiochromic film EBT. At present, the uncertainties associated to many of those variables are unclear. Further investigation will be performed to evaluate the combined total uncertainty for the experiment.

**Table 1:** Some variables that may affect measurement reproducibility

**Categories**	**Variables**
Dose calibration in solid water phantom	PDD calibration (0.8% uncertainty [30])
and beam calibration	Ionizing chamber dosimeter calibration (0.6% uncertainty [30])
	Beam radiation calibration (1.4% uncertainty [30])
Radiochromic film conditions	Differences in lots or sheets of film
	Film manipulation
	Environmental conditions (manipulation and storage)
Calibration film irradiation	Size and number of samples
	Film placement in the water phantom
	Beam orientation and isocenter positioning
Scanning procedure	Post-irradiation waiting period
	Film batch
	Environmental conditions during scan
	Multiple scan passes count
	Scanner type and equipment conditions
	Resolution of the scanned image
Physical phantom	Homogeneity and heterogeneity of the equivalent tissues and tumor
	Positioning and repositioning of the phantom on the irradiation table
	and support
	Positioning of the affixed external reference points
	Film affixing inside the synthetic brain and tumor
Phantom Tomography	Phantom positioning and repositioning
	Reference points
	Image capture and resolution
Calibration and curve fitting	Film set count / dataset size
	Choice of calibration method / equation
	Adjustment method
Experimental irradiation measurements	Irradiation procedure
	Reproducibility of LINAC conditions
TPS planning	Mathematical method
	Dose image representation
	Inhomogeneity in CT application
Image processing	Discrepancies in image scaling
	Resolution, contrast, uniformity of a region of interest (ROI)
	Noise
	Imaging and treatment of coordinate coincidences

The determination of absorbed dose in water with the 15 MV beam, the uncertainties in the different physical quantities, and procedures that contribute to the dose determination shall consider uncertainties up to the calibration of the reference dosimeter at the standards laboratory and the uncertainties associated with the measurements at the reference point in a water phantom. The uncertainties in the various steps are combined in quadrature, yielding the combined standard uncertainty for the determination of the absorbed dose to water at the reference point. The uncertainties in the calibration of the 15 MV beam were estimated as 1.5%. Regarding the uncertainties on the PDD, those are included on the beam calibration, which is estimated as 0.8%. Therefore, an estimated value of 2.3% uncertainty was assumed.

It was possible to verify absorbed doses deposited on the synthetic tumor target, validating the conformal 3D, three-field, T-shaped, radiotherapy protocol. The physical phantom, including the GBM simulator positioned at the center of the brain, was essential to performing the dosimetry. Dosimetric findings showed how relevant was the physical anthropomorphic and anthropometric phantom to our studies, as compared to water phantoms. The latter are made of homogeneous material and do not reproduce mass attenuation deviation from water due to different structures, such as skull and brain, as well as deviation on tissue densities of the physical head and neck phantom. Those variations may introduce uncertainty on dose, particularly on interfaces where electronic equilibrium is disturbed.

The physical head and neck phantom may be disassembled and its brain manipulated in order to insert dosimeters. Positioning of the film-based dosimeters inside the brain structure and the synthetic tumor were adequate, *i. e.* with no damage to the skull or to the rest of the phantom structures. Radio opaque reference points were essential to lining up during planning, irradiation and dose analysis. Synthetic anatomic structures as the brain and the skull were identified on the CT images, demonstrating their radiological equivalency. The tumor simulator presented an heterogeneous physical aspect equivalent to a real GBM.

The physical phantom with synthetic tumor, the radiotherapy planning and the irradiation protocol in a T-shaped pattern at Luxemburgo Hospital in GBM cases, all contribute to make this simulation very realistic, allowing measurements with dosimeters based on EBT2 film.

Future studies shall include several diffuse tumor volumes placed into the brain, with varying sizes and locations, simulation of elements such as edema and hemorrhages, distinct tumor densities and inhomogeneous characteristics, or distinct radiation protocols and beam spectra.

## Conclusion

We investigated EBT2 film irradiation with head and neck 3D-Conformal Radiotherapy treatment plan using an anthropomorphic and anthropometric head and neck phantom using the gamma analysis criterion of 3mm3% and histogram comparisons. Dosimetric experiments performed on a physical phantom, reproducing the irradiation protocol of 15 *MV* 3D-Conformal Radiation Therapy, T-shaped, allowed spatial dose distribution determination on the GBM simulator. Measured absorbed dose on the simulator achieved 70 to 120% of the prescribed values from radiotherapy planning, and showed stratified hot regions. It was demonstrated that Gafchromic EBT2 film, either on red or on green components, may be used as cross-reference for a dose range from 20 to 240 *cGy*, since both components present comparable dose responses with maximum deviation from 2 to 6% respectively, however, they are dependent variable. Dose deviation percentage results indicate almost all points within 1-3% and 3-5% deviation intervals, and a few in the 9-11% interval. Few dark spots in the film caused the appearance of outlier points in the 13-15% interval of dose deviation. On the other hand, DVH results show that the tumor received a homogeneous dose: 100% of the volume received 82% of the maximum dose. No volume received higher dose than 90% of the measured dose. The remainder of the measured DVH, 90% to 100%, is due to those isolated, higher dose dark spots. 83.2% of the points with a gamma value of less than 1 (3%/3mm) for TPS and experimental values, respectively. At the tumor, measured at coronal section, a few dark spots in the film caused the appearance of outlier points in 13-15% of dose deviation percentage. And, as final conclusion, such dosimeter choice and the physical anthropomorphic and anthropometric phantom provided an efficient method for validating radiotherapy protocols.

## Competing interests

The authors declare that they have no competing interests.

## Authors’ contributions

LT - contributed to the preparation of the phantom experiments with films by deciding the location and how films would be placed within the phantom, by handling the films before, during and after the experiment, and scanning and storing films for the collection and analysis of data. She was responsible for writing the article. HGD - contributed during exposure of the phantom in the linear accelerator and on the water phantom calibration, as well as in the interpretation of results and answering questions about exposure technique and the films. TPRC - helped in coordinating the research work and analysis of the results. He was responsible for revising the article. All authors read and approved the final manuscript.

## References

[B1] Wiki de Neurocirugia Glioblastoma multiforme 2012 [http://www.neurocirurgia.com]

[B2] Silker ML Donahue BR Vogelbaum MA Tome WA Gilbert MR Mehta MP Halperin EC, Perez CA, Brady LW Primary intracranial neoplasms Principles and Practice of Radiation Oncology 2008 Lippincott: Williams & Wilkins 730 732

[B3] Dzik C Tumores do sistema nervoso central 2012 [http://www.einstein.br]

[B4] Society AC What are the key statistics about brain and spinal cord tumors? 2012 [http://www.cancer.org]

[B5] Hiesiger EM Hayes RL Pierz DM Budzilovich GN Prognostic relevance of epidermal growth factor receptor (EGF-R) and c-neu/erbB2 expression in glioblastomas (GBMs) J Neurooncol 1993 16 2 93 104 10.1007/BF01324695 7507162

[B6] Kleihues P Ohgaki H Primary and secondary glioblastomas: from concept to clinical diagnosis Neuro-Oncol 1999 1 1 44 51 11550301 10.1093/neuonc/1.1.44 PMC1919466

[B7] Puzzilli F Ruggeri A Mastronardi L Di Stephano D Lunardi P Long-term survival in cerebral glioblastoma. Case report and critical review of the literature Tumori 1998 84 1 69 74 9619719 10.1177/030089169808400115

[B8] Stark AM Nabavi A Mehdorn H Blömer U Glioblastoma multiforme: report of 267 cases treated at a single institution Surg Neurol 2005 63 2 162 169 10.1016/j.surneu.2004.01.028 15680662

[B9] Mineo JF Bordron A Baroncini M Ramirez C Maurage CA Blond S Dam-Hieu P Prognosis factors of survival time in patients with glioblastoma multiforme: a multivariate analysis of 340 patients Acta Neurochir (Wien) 2007 149 3 245 252 10.1007/s00701-006-1092-y 17273889

[B10] Fiveash JB Nordal RA Market JM Ahmed RS Nabors LB Gunderson L, Tepper J High-grade gliomas Clinical Radiation Oncology 2007 Philadelphia: Churchill Livingstone 515 516

[B11] Flickinger JC Niranjan A Halperin EC, Perez CA, Brady LW Stereotactic radiosurgery and radiotherapy Principles and Practice of Radiation Oncology 2008 Lippincott: Williams & Wilkins 381 381

[B12] Biswas T Okunieff P Schell MC Smudzin T Pilcher WH Bakos RS Vates GE Walter KA Wensel A Korones DN Milano MT by T MM Stereotactic radiosurgery for glioblastoma: retrospective analysis Radiat Oncol 2009 4 11 10.1186/1748-717X-4-11 PMC2662864 19292912

[B13] Beckford M Brain tumor survival times double in 30 years 2012 [http://www.telegraph.co.uk]

[B14] Xu XG Xu XG, Eckerman KF Computational phantoms for radiation dosimetry: a 40-Year history of evolution Handbook of Anatomical Models for Radiation Dosimetry 2010 Boca Raton: Taylor & Francis 3 5

[B15] Balmaceda CM Fine RL Merritt HH, Rowland LP Gliomas Merritt Tratado de Neurologia 2000 Rio de Janeiro: Guanabara Koogan 275 280

[B16] Van Battum LJ Hoffmans D Piersma H Heukelom S Accurate dosimetry with Gafchromic EBT film of a 6MV photon beam in water: what level is achievable? Med Phy 2008 35 2 704 716 10.1118/1.2828196 18383692

[B17] Lindsay P Rink A Ruschin M Jaffray D Investigation of energy dependence of EBT e EBT2 Gafchromic film Med Phy 2010 37 2 571 577 10.1118/1.3291622 20229865

[B18] Arjomandy B Tailor R Anand A Sahoo N Gillin M Prado K Vicic M Energy dependence and dose response of Gafchromic EBT2 film over a wide range of photon, electron, and proton beam energies Med Phy 2010 37 5 1942 1948 10.1118/1.3373523 20527528

[B19] Arjomandy B Tailor R Zhao L Devic S EBT film as a depth-dose measurement tool for radiotherapy beams over a wide range of energies and modalities Med Phy 2012 39 2 912 921 10.1118/1.3678989 22320801

[B20] GAFCHROMIC®; EBT2 Self-developing Film for Radiotherapy Dosimetry 2009 Alps Road Wayne, NJ: ISP international specialty products/advanced materials: a business unit of ISP

[B21] Thompson L Desenvolvimento de um fantoma antropomórfico e antropométrico de cabeça e pescoço infanto-juvenil e de um fantoma computacional para estudo radiodosimétricos em câncer de laringe e faringe PhD thesis Federal University of Minas Gerais, Nuclear Engineering Department 2004

[B22] Schettini MP Maia M Campos TPR The development of an anthropomorphic and anthropometric thorax female phantom for experimental radiodosimentry Int J Low Radiat 2007 4 124 135

[B23] Thompson L Campos TPR A head and neck simulator for radiology and radiotherapy IEEE Trans Nuclear Sci 2013 PP 1 9

[B24] Low D Harms W Mutic S Purdy J A technique for the quantitative evaluation of dose distributions Am Assoc Phys Med 1998 25 5 656 661 10.1118/1.598248 9608475

[B25] Depuydt T Esch A Huyskens D A quantitative evaluation of IMRT dose distributions: refinement and clinical assessment of the gamma evaluation Radiother Oncol 2002 62 3 309 319 10.1016/S0167-8140(01)00497-2 12175562

[B26] Butson M Cheung T Yu P Radiochromic film dosimetry in water phantoms Phys Med Biol 2001 46 N27 N31 10.1088/0031-9155/46/1/405 11197685

[B27] Albertini F Casiraghi M Lorentini S Rombi B Lomax A Experimental verification of IMPT treatment plans in an anthropomorphic phantom in the presence of delivery uncertainties Phys Med Biol 2011 56 14 4415 4431 10.1088/0031-9155/56/14/012 21709345

[B28] Nakano M Hill R Whitaker M Kim J Kuncic Z A study of surface dosimetry for breast cancer radiotherapy treatments using Gafchromic EBT2 film Appl Clin Med Phys 2001 13 3 83 97 10.1120/jacmp.v13i3.3727 PMC5716557 22584169

[B29] Horsfield M Gafchromic EBT2 film dosimetry in an anthropomorphic head and neck phantom measuring Intensity Modulated Radiation Therapy (IMRT) dose distributions PhD thesis University of Wollongong, Department of Engineering Physics 2012

[B30] Andre P Burns D Hohlfeld K Huq M Kanai T Laitano F Smyth V Vynckier S Absorbed dose determination in external beam radiotherapy: an international code of practice for dosimetry based on standards of absorbed dose to water. R: IAEA TRS-398 Int At Energy Agency IAEA 2004 11 b 13 181

[B31] Devic S Seuntjens J Sham E Podgorsak EB Schmidtlein CR Kirov AS Soares GC Precise radiochromic film dosimetry using a flat-bed document scanner Med Phy 2005 32 7 2245 2253 10.1118/1.1929253 16121579

[B32] 2D/3D I Digitizing software 2012 [http://www.imagedig.com/]

[B33] Butson MJ Yu PKN Cheung T Metcalfeb P Radiochromic film for medical radiation dosimetry Mater Sci Eng: R Rep 2003 2003 41 3–5 61 120

[B34] OriginLab Origin and originPro - data analysis and graphing 2012 [http://www.originlab.com/]

[B35] Médica MI CAT3D - Sistema para planejamento de Radioterapia Tridimensional Conformada e IMRT 2013 [http://www.mevis.com.br/index.php?cat3d-1]

